# Localized disorganization of the cochlear inner hair cell synaptic region after noise exposure

**DOI:** 10.1242/bio.038547

**Published:** 2018-11-30

**Authors:** Anwen Bullen, Lucy Anderson, Warren Bakay, Andrew Forge

**Affiliations:** UCL Ear Institute, London, UK WC1X 8EE

**Keywords:** Inner hair cell, Noise exposure, Pre-synaptic, Vesicle

## Abstract

The prevalence and importance of hearing damage caused by noise levels not previously thought to cause permanent hearing impairment has become apparent in recent years. The damage to, and loss of, afferent terminals of auditory nerve fibres at the cochlear inner hair cell has been well established, but the effects of noise exposure and terminal loss on the inner hair cell are less known. Using three-dimensional structural studies in mice we have examined the consequences of afferent terminal damage on inner hair cell morphology and intracellular structure. We identified a structural phenotype in the pre-synaptic regions of these damaged hair cells that persists for four weeks after noise exposure, and demonstrates a specific dysregulation of the synaptic vesicle recycling pathway. We show evidence of a failure in regeneration of vesicles from small membrane cisterns in damaged terminals, resulting from a failure of separation of small vesicle buds from the larger cisternal membranes.

## Abstract (precis)

**Summary:** Hearing deficits after noise exposure are linked to afferent terminal loss. We show that afferent terminal loss is concomitant with changes to inner hair cell pre-synaptic ultrastructure indicating vesicle-recycling deficits.

## INTRODUCTION

Inner hair cells (IHC) are the principal receptor cells in the mammalian cochlea. They detect fluid motion induced by sound vibrations and transduce it into neural impulses that are relayed to the brain. Projecting from the hair cell's apical surface is an organised bundle of microvillus-like stereocilia, deflection of which in response to sound vibrations modulates a current flow through the hair cell. The resultant changes in hair cell polarisation are coupled to regulated release of glutamate neurotransmitter at the synapses between the IHC and the terminals of auditory nerve fibres (ANF) that form synapses around the IHC's basolateral membrane. The presynaptic (IHC) side of the synapse is characterised by a synaptic ribbon, a rounded or linear structure to which neurotransmitter vesicles are tethered and thereby held close to the presynaptic membrane (Matsubara et al., 1996). This is thought to enable the rapid and prolonged (indefatigable) release of neurotransmitter onto the clusters of AMPA-type glutamate receptors on the opposing post-synaptic membrane with the consequent continuous and persistent neural stimulation necessary for perception of auditory information.

Each IHC is innervated by several (up to ∼20) different ANF, but an individual ANF contacts only one IHC (Liberman et al., 1990). Physiological recording from individual fibres suggests there are sub-types of ANF innervating an individual IHC. Some have low spontaneous rates (SR) of firing and high thresholds of stimulation; others have high spontaneous firing rates and low sensitivity thresholds. These different fibre types are thought to provide a means to encode loudness: low-threshold fibres would be stimulated by quieter sounds (low thresholds) and the high-threshold fibres would be stimulated only as sounds became louder. The accurate functional regulation whereby graded modulation of current flow through the cell enables delivery and continuous replenishment of neurotransmitter at the appropriate terminals at the right time suggests a structural organisation of the IHC to support it. Our previous work using high resolution 3D imaging (Bullen et al., 2015) did indeed show that internal sub-cellular organisation of mitochondria and internal membranes of the IHC corresponds to the innervation pattern, indicating that hair cell ultrastructure and neuronal organisation may be linked. Disruption of the cellular organisation could have consequences for auditory perception.

Exposure to high noise levels causes damage to, and loss of hair cells, resulting in permanent decrements in hearing sensitivity as measured by audiometric thresholds [permanent threshold shift (PTS)] (Puel et al., 1998). After exposure to damaging noise a proportion of the afferent boutons of ANFs swell dramatically and then apparently degenerate (Robertson, 1983). There is strong evidence that this swelling is caused by glutamate excitotoxicity (Puel et al., 1998). Damage mostly occurs in the higher frequency regions of the cochlea, more basal along the cochlear spiral than the frequency place of the original noise exposure. In mice, for example, a noise exposure between 8–16 kHz causes maximal neuronal damage at frequency regions above 32 kHz (Kujawa and Liberman, 2009).

Milder noise exposures cause an initial elevation in auditory thresholds that subsequently recover to pre-exposure levels (temporary threshold shift, TTS). However, it is now clear that exposure conditions that result in TTS still cause permanent damage to ANFs in the absence of any loss of hair cells (Furman et al., 2013; Kujawa and Liberman, 2009). Predominantly only a subset of ANF fibres, those with low spontaneous rates (LSR), high thresholds and wide dynamic ranges (Costalupes et al., 1984; Furman et al., 2013) are affected. While some studies suggest loss of most terminals is permanent (Furman et al., 2013; Kujawa and Liberman, 2009; Lin et al., 2011), others have found evidence indicating regeneration of some synapses (Ruel et al., 2007; Shi et al., 2015, 2013), but regenerated synapses may function abnormally, with longer latencies and reduced compound action potentials, and there may also be abnormal function in those high spontaneous rate fibres that remain after noise exposure (Shi et al., 2016; Song et al., 2016). The continuing permanent neuronal deficits may be responsible for a variety of subtle hearing deficiencies, a condition that has become known as ‘hidden hearing loss.’ (Kujawa and Liberman, 2015; Schaette and McAlpine, 2011).

The lack of IHC degeneration under conditions that produce TTS has been taken as evidence that the noise exposure affects only the sub-population of ANFs with no or minimal effects on the IHCs themselves. However, concomitant with damage to the afferent boutons of ANFs, changes also occur to the synaptic ribbons of IHCs. Changes to their size and location, the presence of multiple ribbons, and a decrease in the number of ribbon-attached vesicles, have all been reported after noise exposure (Ruel et al., 2007; Shi et al., 2016, 2013). This may indicate that there are effects of noise exposure upon the IHCs themselves that have not been appreciated previously. It may be that the effects of the noise exposure disrupt the sub-cellular organisation of the IHC that we previously demonstrated. To address this question, we used high resolution 3D imaging to examine IHCs four weeks after a TTS noise exposure. We identified changes in whole cell morphology, determined the pattern of loss of afferent terminals, and described ultrastructural changes with potential functional consequences in the pre-synaptic regions adjacent to terminals showing evidence of damage.

## RESULTS

### Noise exposed cells show significant changes to cell shape, but not organelle distribution

**Fig. 1.**
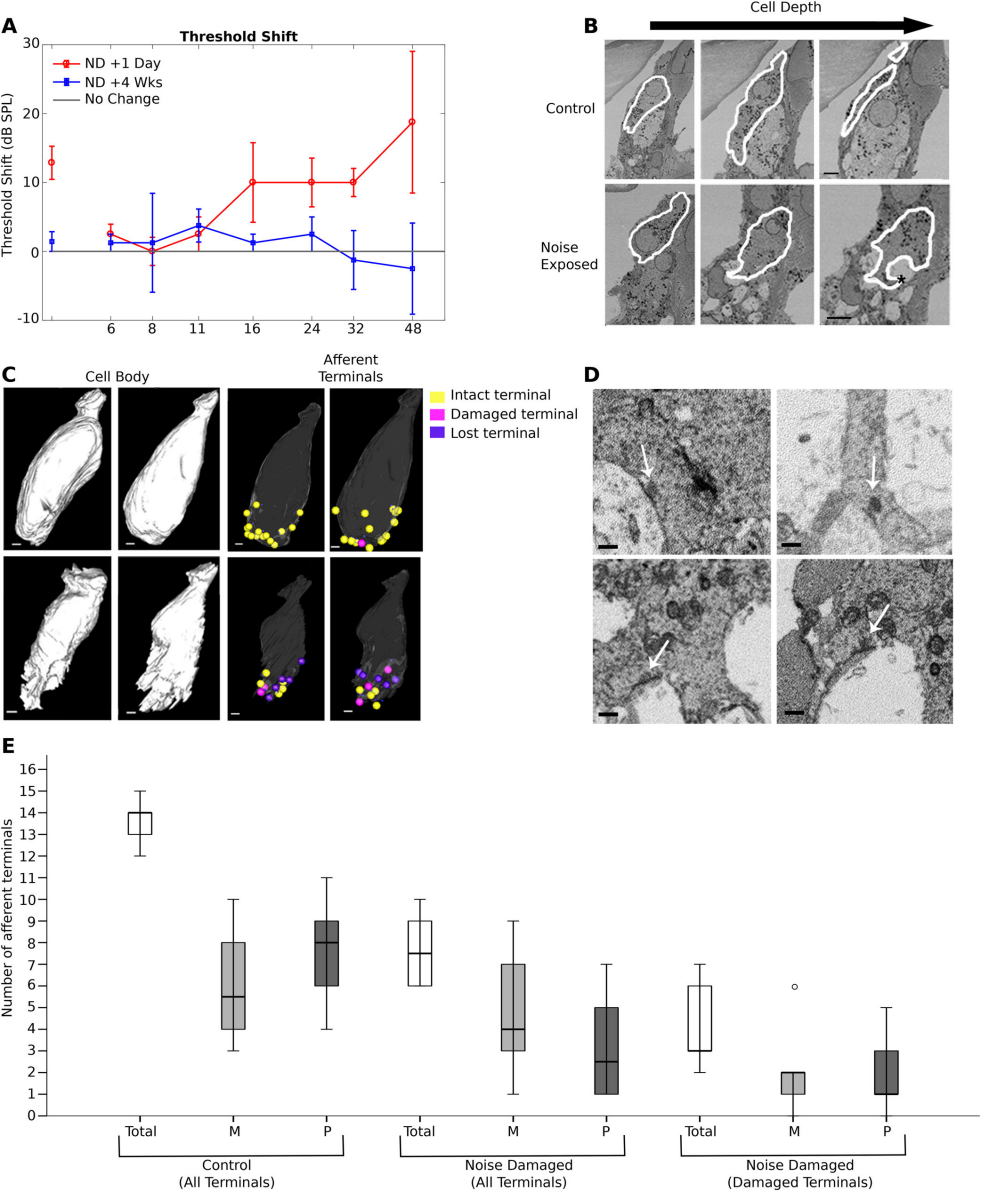
**Threshold shifts in ABR as result of noise exposure.** Changes to whole cell morphology and afferent terminals after noise damage. (A) Threshold shift as a result of octave-band noise exposure as measured from ABRs. A threshold shift at frequencies ≥16 kHz is present 1 day (solid red line, circular symbols) after noise exposure, but resolves after 4 weeks (green line, triangular symbols). Grey line indicates no change. Whole cell analysis from SBF-SEM images. (B) Slices from SBF-SEM image stacks for two control and two noise damaged inner hair cells. Arrow indicates increasing depth through the cell. White lines show contour tracing of the cytoplasmic membrane. (*) Indicates example of the vacuolation occurring beneath the cells in noise exposed animals. (C) Reconstructed cell bodies and afferent terminal positions from the cytoplasmic membrane contours. The distortion of noise damaged cells caused by afferent terminal expansion can be clearly seen. Afferent terminal positions on control and noise damaged cells, represented by spheres. (Yellow) Intact terminals, (pink) damaged terminals, (purple) lost terminals. (D) Examples of synaptic ribbons and postsynaptic densities observed in SBF-SEM datasets, top pair: intact terminals, bottom pair: damaged terminals. (E) Number afferent terminals in modiolar and pillar cell facing hemispheres. There were no significant differences in control cells or in noise-exposed cells in the total population of terminals (*P*=0.292 control, 0.429 noise exposed). [Two-tailed Mann–Whitney U. *n*=6 cells from one (control) or two (damaged) animals. Error bars represent mean±s.d.] Scale bars: (B–C) 2 µm.

Auditory threshold, the lowest sound pressure level that evokes a neural response, can be estimated from recordings of the auditory brainstem response (ABR) that reflects the neural activity evoked by an auditory stimulus. Previous work has shown that a 2-h exposure to 100 dB SPL, octave-band noise induces a TTS (Hesse et al., 2016). Using this regime, ABRs were measured in individual mice immediately before, 1 day after, and 4 weeks after noise exposure. One day after exposure, animals exhibited increased ABR thresholds in response to broadband clicks and pure tone frequencies ≥16 kHz (Fig. 1A). Four weeks later in the same animals ABR thresholds to clicks and to all frequencies tested had recovered such that they no longer differed from the pre-exposure values. The raised thresholds followed by recovery to pre-exposure levels indicated a temporary threshold shift had occurred in all animals tested. After the second post-noise exposure ABR recording cochleae were removed and prepared for electron microscopy. Mid-modiolar sections through the noise damaged region were cut for serial block face-scanning electron microscopy (SBF-SEM) and for electron tomography.

3D reconstructions of the cell bodies of the IHCs were obtained from the SBF-SEM image sets following segmentation by tracing the cell body (Fig. 1B, white lines). IHCs are typically asymmetric flask-shaped cells with one side more rounded than the other (see Fig. 1C control cells). After 4 weeks recovery following the noise exposure, the IHCs in noise-exposed animals were severely deformed by swelling at the locations of afferent terminals, with cells showing indentations and shape changes in the basolateral regions compared to control cells (Fig. 1C). Despite the greater convolutions of the distorted membrane, the surface area of noise-exposed cells was reduced compared to control cells (mean control: 1394.59 µm^2^±23.28 noise damaged: 1217.8 µm^2^±59.94 *n*=6 cells in each case). Some of this difference may be accounted for by the difficulty of following the convoluted cytoplasmic membranes of the noise damaged cells through the image stack, but the length of the cell from the its apex (measured from the plasma membrane at the cell's apex) to the base (the lowest point of the plasma membrane) was similar in the two groups (mean control: 38.31 µm±0.82 noise damaged: 39.77 µm±1.81), showing that the full profile of the cell was segmented. Despite the severe changes to the shape of the cells, the distribution of organelles in the infranuclear region did not appear to be affected. In our previous work (Bullen et al., 2015) we identified by point counting stereology an asymmetric arrangement of the infranuclear endoplasmic membranes and mitochondria in IHCs, with these organelles clustered either on the side of the IHC facing the supporting pillar cell (the pillar face) or on the side facing the central axis of the cochlea (the modiolar face). Application of the same point counting stereology to the infranuclear region of IHC in the present work showed a similar asymmetric organisation in both control and noise-exposed cells. To assess whether this structural asymmetry of the cells was significant, the side with the highest number of stereology points classified as membranes or mitochondria was compared to the side with the lowest. Both control and noise exposed cells showed significant differences in the number of points classified as membranes or mitochondria between the two sides (control *P*=0.009, noise exposed *P*=0.002) demonstrating that organelle distribution was asymmetric in both groups.

### Damage to afferent terminals does not predominantly affect one side of IHCs

The position of afferent terminals around the hair cells was also modelled using representative spheres of the same size for all terminals (Fig. 1C). Afferent terminals were identified by the presence of a bouton adjacent to the basolateral membrane of the IHC in which mitochondria and cytoplasmic density were evident and there was a density at the synaptic membranes apposing a synaptic ribbon in the IHC (examples in Fig. 1D). On noise-exposed cells, some boutons appeared as intact afferent terminals, no different from those seen in controls. These were classified as ‘intact’ boutons. Other bouton-like indentations were enlarged swellings containing no obvious cytoplasmic material in which there were membranous vacuoles but a synaptic density facing a synaptic ribbon were both evident. These were classified as ‘damaged’ boutons (Fig. 1B, asterisk). Swollen boutons were occasionally observed on control cells, but represented <5% of all terminals examined. In contrast swollen afferent terminals made up 55% of terminals present on noise damaged cells. It was also observed in cochlea sections from noise damaged animals that the severe bouton swelling observed did not appear to extend to regions apical and basal of the expected damage region. Noise-damaged cells also had a proportion of bouton-like indentations adjacent to the IHC plasma membrane, which were similar in shape to damaged boutons and enclosed membranous vacuoles, but did not show a synaptic density nor a recognisable apposing synaptic ribbon in the IHC. The total number of boutons and bouton-like indentations of these three identified types around the body of a noise-exposed IHC was 12.17±0.75, little different from 13.67±0.42 afferent terminals around an IHC in a control animal not exposed to noise, demonstrating that the ‘empty’ bouton-like indentations were sites where the afferent terminal had completely degenerated. It has been shown that a proportion of postsynaptic densities and ribbons are lost after noise damage (Kujawa and Liberman, 2009). Three classes of ‘terminals’ were therefore identified in the noise-exposed IHC (Fig. 1C): ‘intact’ (yellow), ‘damaged’ (pink) and ‘lost’ (purple). The mean number of intact and damaged terminals on noise-damaged cells was 7.83±0.60 compared with 13.67±0.42 in the unexposed control cells (Fig. 1E), indicating that despite recovery in auditory threshold there was a significant loss of terminals following noise exposure. Additionally, of the remaining terminals where both a synaptic density and a pre-synaptic ribbon were retained, ca. 55% were damaged. These findings suggest that it is possible to maintain auditory thresholds with fewer than 30% of IHC afferent terminals intact.

It has been shown in cats that high SR fibres synapse to the pillar face and the modiolar face of the cell, but low and medium SR fibres synapse predominantly to the modiolar face (Liberman, 1982; Liberman et al., 1990). As low SR fibres have been shown to be particularly susceptible to noise damage (Furman et al., 2013) the loss of terminals around the pillar and modiolar hemispheres was assessed. There was no significant difference between the numbers of afferent terminals on each side in control cells or in noise-exposed cells (Table 1). There was also no significant difference between the modiolar and pillar sides in the mean number of ribbon synapses present on damaged terminals and this persisted even when lost terminals were included in the analysis (Table 1). When only lost terminals were included in the analysis the difference between the two sides approached significance (*P*=0.063, Table 1). These results suggest that damage to the synapses was not preferentially affecting one side of the cell in this sample, although a greater number of lost terminals (i.e. those without evident ribbons and post synaptic densities) was present on the modiolar side.

**Table 1.**
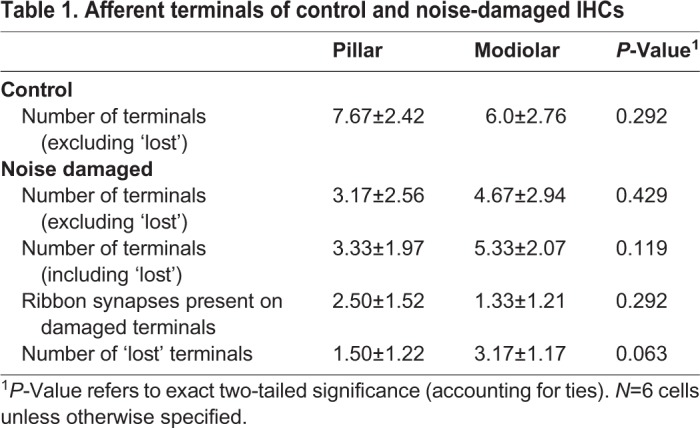
**Afferent terminals of control and noise-damaged IHCs**

### Structural characterisation of the IHC pre-synaptic region reveals differences between intact and noise-damaged terminals

As the stereological analysis described above had shown that severe disruption to IHC shape in the noise-damaged region did not disturb the overall intracellular organisation of membrane systems and mitochondria inside the cell, the possibility of localised effects at synaptic regions of the IHC was explored. Disruption of synaptic ribbons and functional changes in repaired synapses has been described previously (Ruel et al., 2007; Shi et al., 2016, 2013). Electron tomography was therefore performed to obtain high-resolution 3D images of ultrastructural organisation to examine the effects on ribbons and the pre-synaptic regions of the IHC concomitant with terminal damage. For this analysis only regions where there was a synaptic density at the membrane interface between the terminal and the cell was examined. The regions of the IHC associated with a ‘lost’ terminal were not. Because of the thickness of sections for tomography, where there was no membrane density visible in the section, it was not possible to distinguish between a region of swollen terminal outside the area of the membrane density and a presynaptic region facing a ‘lost’ terminal. Likewise, as synaptic densities are much wider than the width of a synaptic ribbon, the absence of a ribbon in a section could not be taken as an absence of a ribbon from the presynaptic region of the cell.

**Fig. 2.**
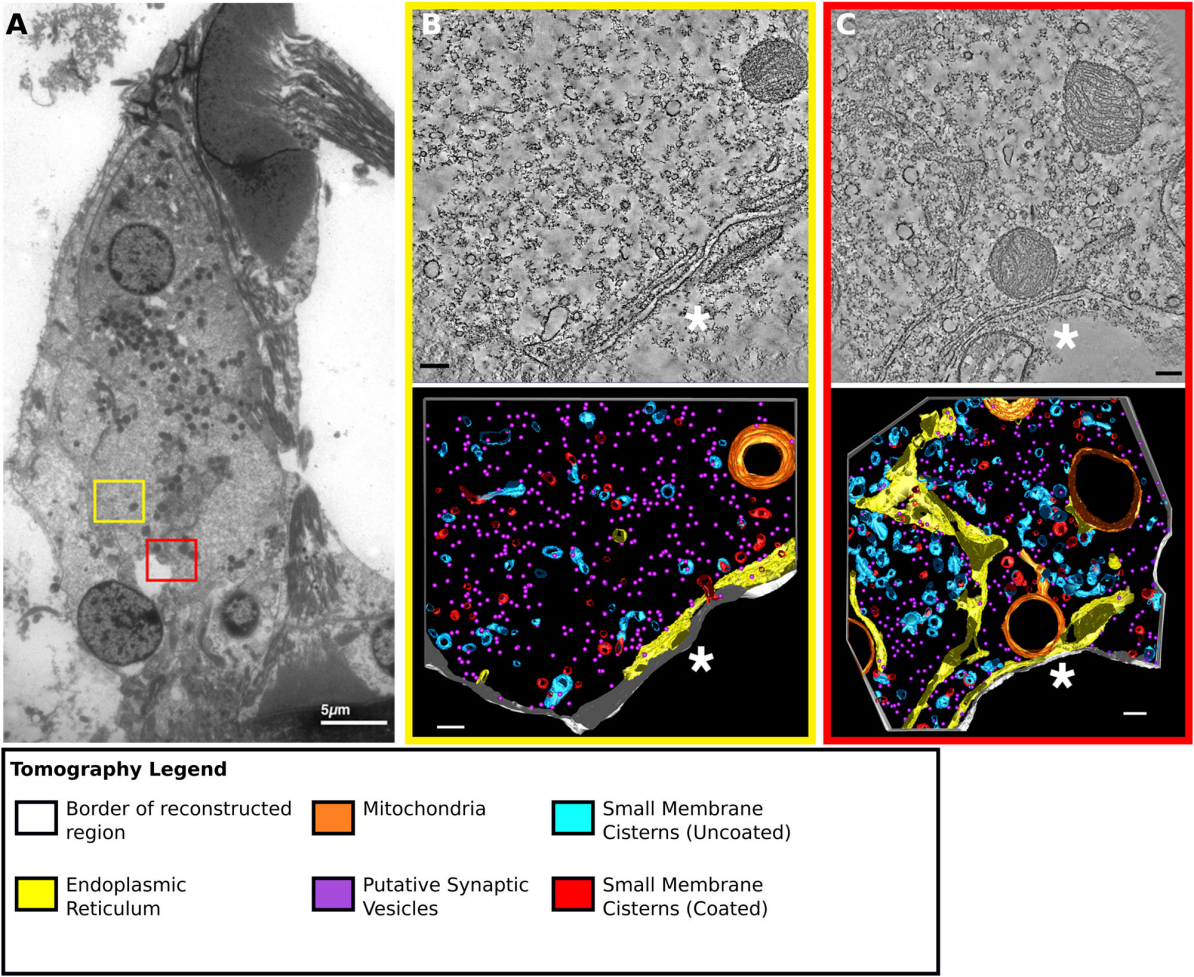
**Intact and damaged terminals on the same cell showed differences in pre-synaptic ultrastructure.** (A) TEM image of IHC from 4–8 kHz noise damaged animal, coloured boxes represent regions from which tomographic data was collected on an intact terminal (B, yellow), and a damaged terminal (C, red). Colours represent: white, border of reconstructed region ‘*’ indicates the presynaptic membrane; orange, mitochondria; yellow, endoplasmic reticulum; light blue, small membrane cisterns (uncoated); red, small membrane cisterns (coated); light-blue/red, mixed membranes; purple spheres (small), putative synaptic vesicles; violet, synaptic ribbon; pink, other endosome. Despite the close proximity, these terminals had very different morphologies. Scale bars: (A) 5 µm, (B–C) 200 nm.

**Fig. 3.**
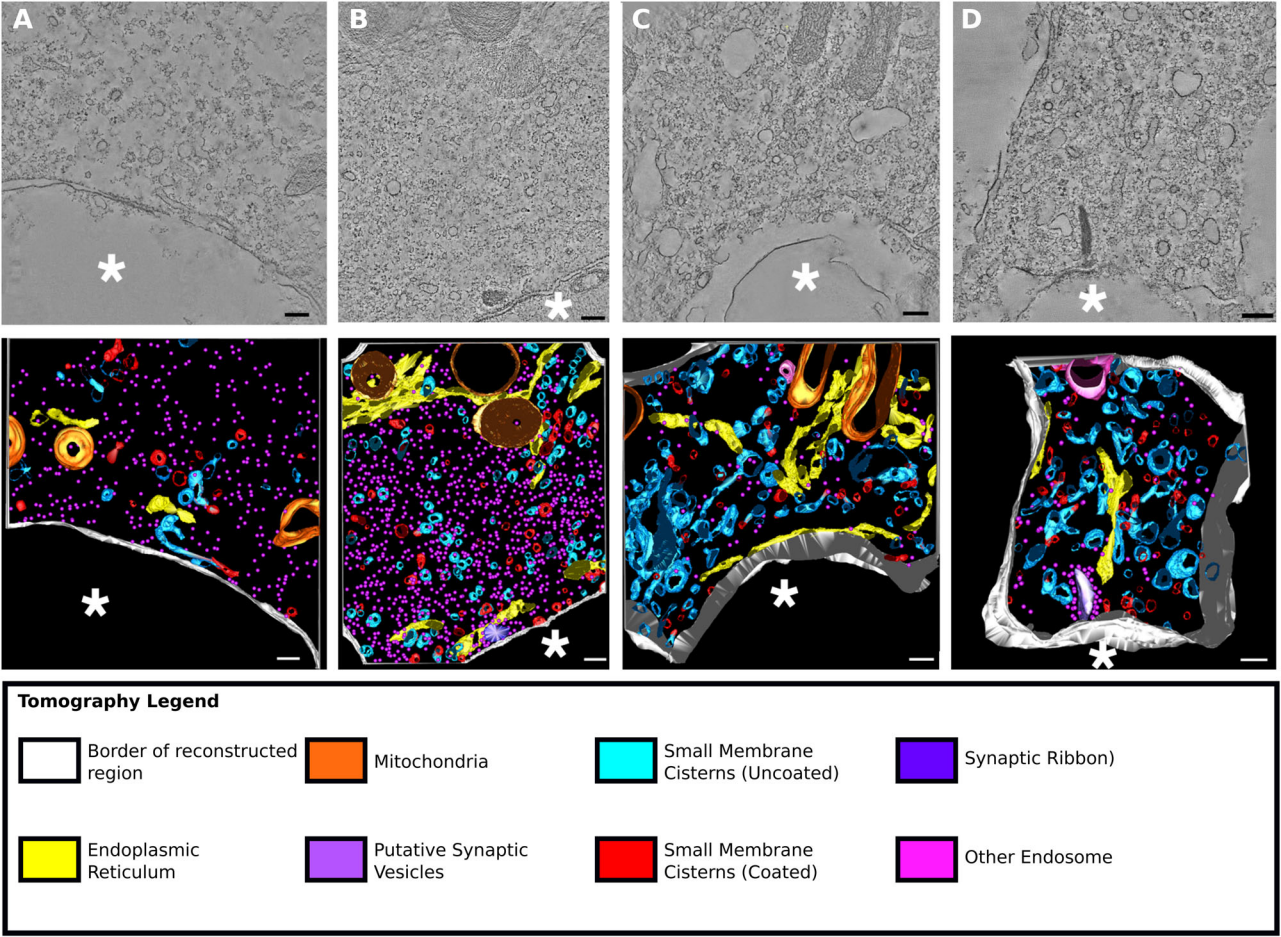
**Pre-synaptic regions adjacent to damaged terminals showed ultrastructural changes compared to control cells.** (A–D) Tomographic slice and reconstruction from pre-synaptic region in control and noise exposed animals. Colours and asterisks as Fig. 2. (A) Control animal (intact terminal), (B) 8–16 kHz noise damaged animal (intact terminal), (C) 4–8 kHz noise damaged animal (damaged terminal), (D) 8–16 kHz noise damaged animal (damaged terminal). Scale bars: (A–D) 200 nm.

Thus, analyses were performed on terminals identified only by the presence of synaptic densities at the interface of the membranes and all terminals with evident synaptic density were imaged. An equal number of pre-synaptic regions from the modiolar and pillar sides of the cells underwent full 3D reconstruction and segmentation in both intact and damaged populations to prevent any potential preference for high or low SR fibres in the analysis. Montage electron tomography was used to produce high resolution 3D imaging of an area of IHC cytoplasm approximately 1 µm^2^ around the synaptic density. These volumes were then segmented and reconstructed to produce 3D models of membranous objects in the pre-synaptic regions (Figs 2,3). Regions with and without ribbons were selected to ensure a survey that encompassed as much of the pre-synaptic region as practical, but the synaptic density was always present. Membranes were colour coded according to their structural properties (see next section and Fig. 3). Mitochondria (orange) and synaptic ribbons (dark purple) were also included in the reconstructions. We noted that some terminals had pre-synaptic subsurface cisternae that appeared to be composed of endoplasmic reticulum-like membranes. Such cisterns have been previously reported in OHC synapses with efferent terminals, where they have been suggested to act as a buffer to isolate calcium signals (Fuchs et al., 2014), and it is possible that they may perform a similar function in inner hair cells at afferent synapses.

Full montage tomographic reconstruction and segmentation of the pre-synaptic regions facing intact terminals were compared with those facing the damaged terminals. The pre-synaptic regions facing intact terminals were drawn from two populations: regions from control cells (*n*=3) (Fig. 3A) and those apposed to intact terminals in noise-damaged cells (*n*=3) (Fig. 3B) from a total of four different animals. Damaged terminals from animals exposed to two different frequencies were examined. The 16 kHz region was examined in animals exposed to 4–8 kHz (*n*=3) (Fig. 3C) and the 32 kHz region in animals exposed to 8–16 kHz (*n*=3) (Fig. 3D) (three different animals). Previous work has indicated that morphology and release kinetics may vary according to frequency (Liberman, 1982; Schnee et al., 2005).

Intact terminals of noise-exposed cells were indistinguishable from those in control (unexposed) cells. This was despite the fact that in the noise-exposed cells intact and damaged terminals were present in the same cell, often in close proximity (Fig. 2A–C). Measured distances (taken from the centre of the synaptic density and measured in XY on a single Z plane) between adjacent damaged and intact terminals on noise damaged cells, taken from six terminals across three noise-damaged animals, ranged between 1.8 µm and 6.9 µm (mean=4 µm±2). These results suggest that effects of damage were closely confined and localised to the pre-synaptic regions facing damaged terminals and did not extend to those of apparently intact terminals in the same cell.

The most striking feature of the pre-synaptic regions apposed to damaged terminals was the accumulation of intracellular membrane, in the form of small membrane cisterns (Fig. 3A–D). Under normal conditions a number of small membrane cisterns exist in the pre-synaptic region. These are thought to act as part of the machinery for recycling of endocytosed membranes into new synaptic vesicles (Kantardzhieva et al., 2013; Lenzi et al., 2002). Membranous structures in the pre-synaptic region were classified into groups according to similarities in structure.

**Fig. 4.**
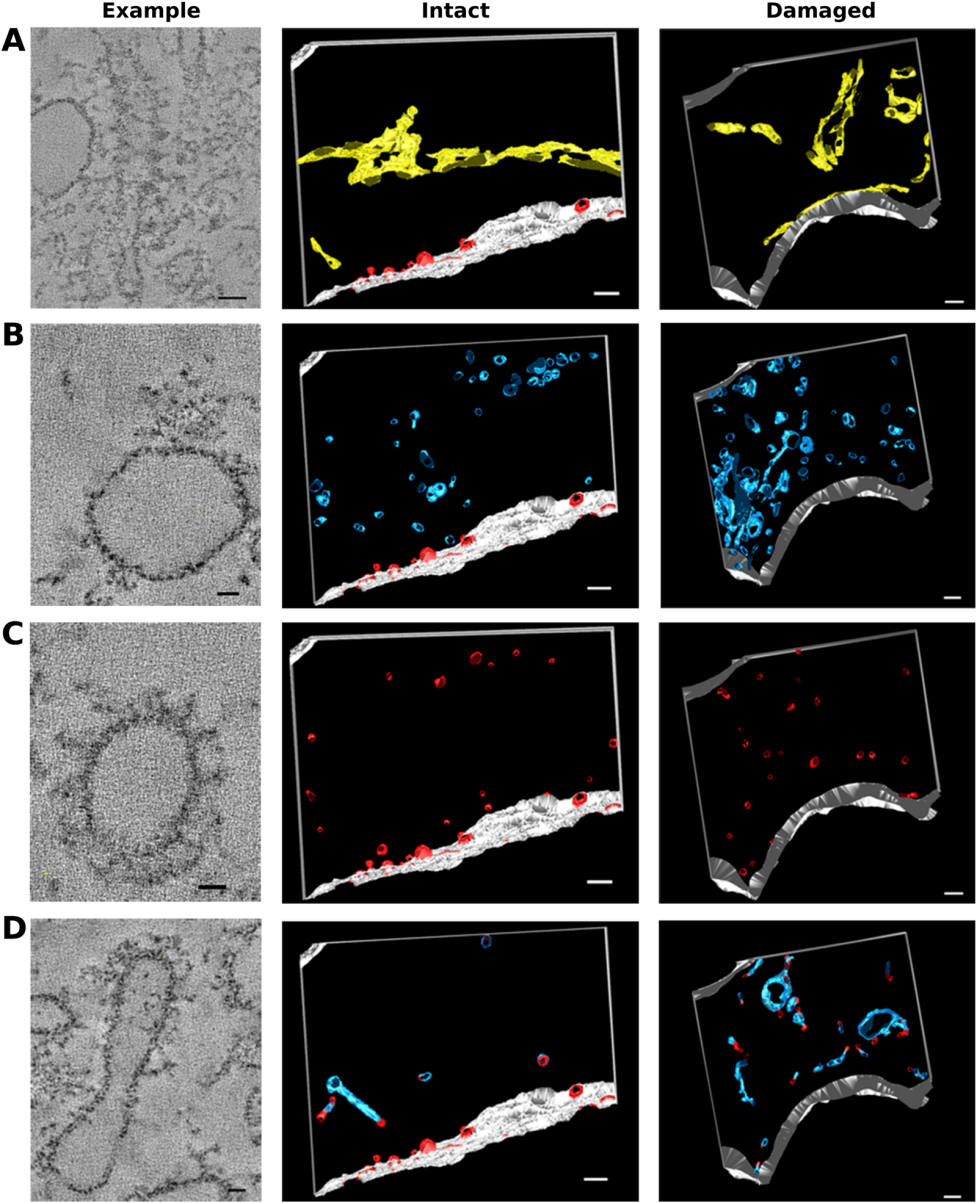
**Uncoated membrane cisterns and cisterns with coated and uncoated regions were both increased in pre-synaptic regions from damaged terminals.** (A–D) Example images and distributions for different membrane populations in pre-synaptic regions of intact and noise damaged terminals. (A) Endoplasmic reticulum. (B) Uncoated membrane cisterns, (C) coated membrane cisterns (red patches on pre-synaptic membrane represent endocytic events), (D) mixed membranes. Scale bars: (A, slice) 50 nm, (B–D, slices) 20 nm, (A–D, reconstructions) 200 nm.

Rough endoplasmic reticulum (yellow in reconstructions) was identified by the presence of ribosomes on the membrane surface, and discernible stained particles inside the membrane lumen (Fig. 4A). Membrane cisterns had no lumen contents and had no ribosomes attached (Fig. 4B–D). Cisterns could be divided into those with smooth membranes (blue) (Fig. 4B) and those with membranes decorated with a coating of approximately L-shaped particles, also seen on areas of the plasma membrane undergoing endocytosis, and putatively identified as clathrin (red) (Fig. 4C). A population of mixed membranes, with some regions smooth and some coated, were also present (Fig. 4D). These three populations were referred to as ‘uncoated membranes’, ‘coated membranes’ and ‘mixed membranes’, respectively. A large number of small (30–60 nm) membranous spheres were present (magenta). These were putatively identified as neurotransmitter vesicles based on their size and similarity (in size and shape) to vesicles attached to the synaptic ribbons (purple) (Fig. 6).

**Fig. 5.**
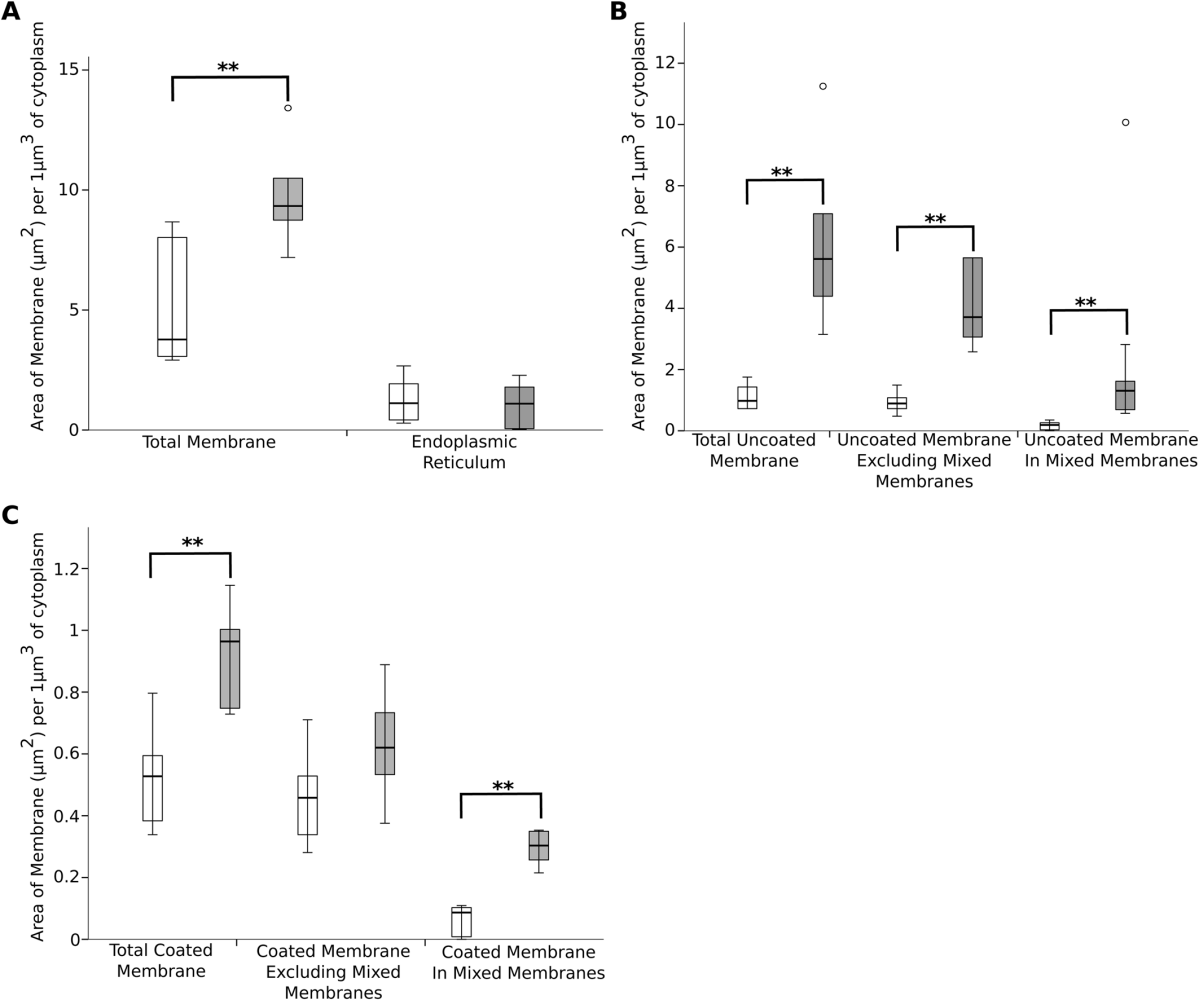
**Significant differences in membrane area for some membrane types between intact and damaged terminals.** White bars represent intact terminal population; grey bars represent damaged terminal population. (A) Total membrane (sum of all populations) was significantly increased in the damaged terminals (*P*=0.009), but the endoplasmic reticulum component was not changed. (B) Uncoated membrane was significantly increased, in both uncoated membrane cisterns (*P*=0.002) and in the proportion forming part of mixed membrane cisterns (*P*=0.002). (C) Coated membrane was increased in total proportion, but this increase was only significant in the proportion forming part of mixed membranes (*P*=0.002), when this was excluded, the proportion of coated membrane cisterns was not significantly different (*P*=0.093). **P=<0.01 [two-tailed Mann–Whitney U. *n*=6 terminals from four (intact) or three (damaged) animals. Error bars represent mean±s.d.].

The amount of membrane present in the pre-synaptic region was calculated by estimation of the surface area of the reconstructed membrane cisterns in the 3D models. Intracellular membrane in the pre-synaptic region was significantly increased in the noise-damaged terminals (Fig. 5A, Table 2). This increase did not occur equally across all of the membrane populations (Fig. 5A–C). The mean area of endoplasmic reticulum was similar in both damaged (shaded bars) and intact (open bars) pre-synaptic regions (Fig. 5A, Table 2). In contrast, uncoated membrane cisterns showed a dramatic increase in combined total area. This area measurement included uncoated membrane that formed part of a mixed membrane. Excluding uncoated membrane area from mixed membranes slightly reduced, but did not abolish, the significant increase in area. The uncoated membrane forming mixed membranes was also significantly increased (Fig. 5B, Table 2).

**Table 2.**
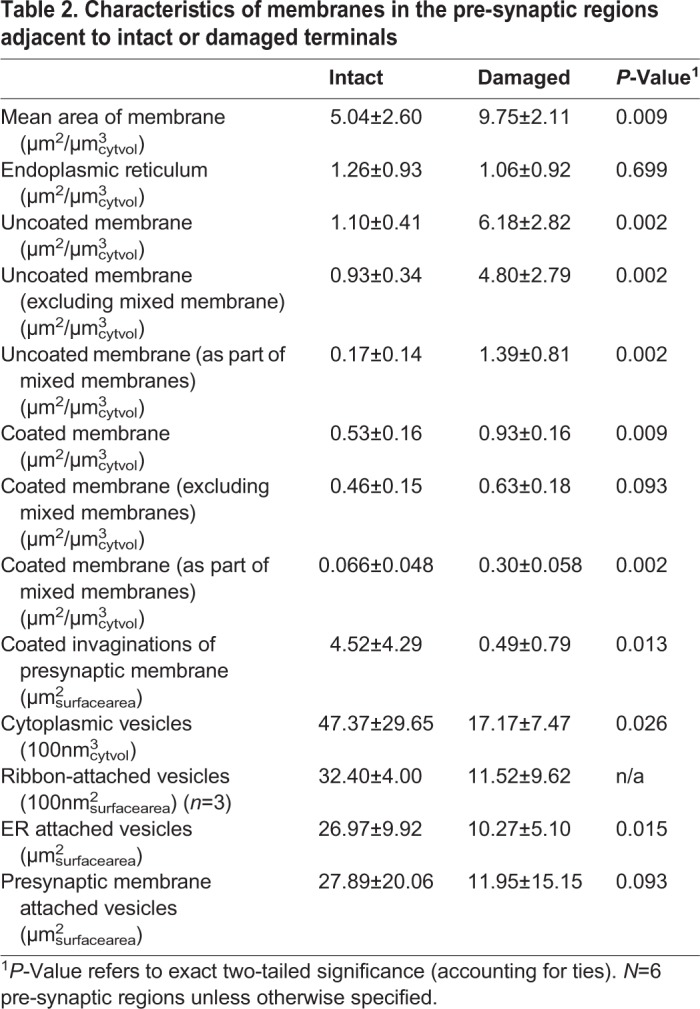
**Characteristics of membranes in the pre-synaptic regions adjacent to intact or damaged terminals**

The total area of coated membrane (inclusive of coated membranes forming part of mixed membranes) showed a slight increase. This difference was reduced when coated membranes from mixed membranes were excluded from the total. The total area of coated membrane contained within the mixed membrane population was much increased in the damaged terminals (Fig. 5C, Table 2). The increase in total coated membrane was therefore mostly occurring in the mixed membrane population, rather than in isolated coated membrane cisterns. In addition, coated invaginations of the pre-synaptic membrane were also reduced in the damaged synapses, suggesting fewer endocytosis events occurring, although these counts had a large variability (Table 2). These results together suggest that in the damaged terminals the processing of endocytosed membrane into synaptic vesicles was not occurring at the same rate as in the intact terminals.

### Pre-synaptic regions of noise damaged terminals contain a reduced number of putative synaptic vesicles

**Fig. 6.**
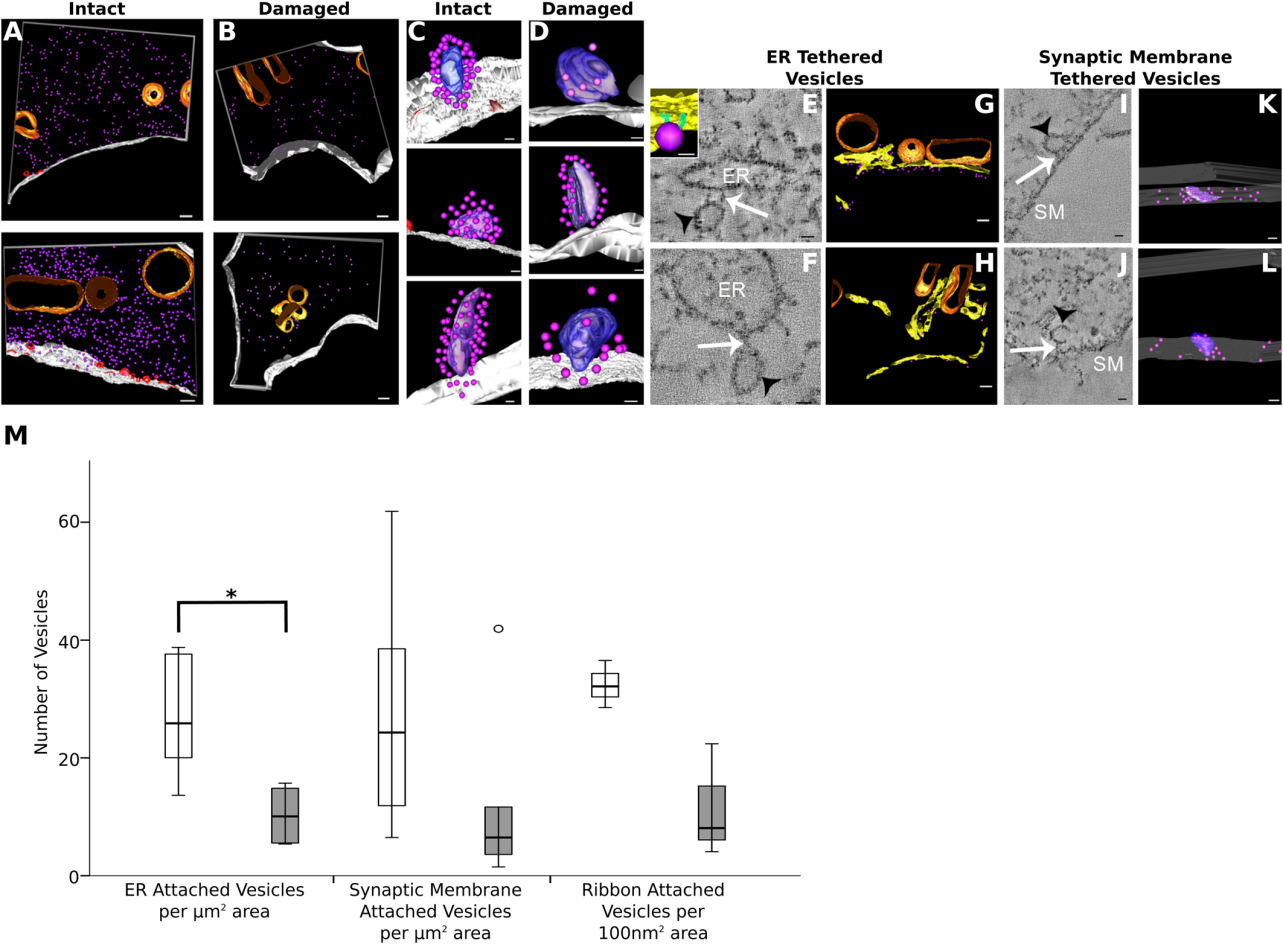
**Synaptic vesicles in pre-synaptic regions were reduced in regions adjacent to damaged terminals.** (A) Cytoplasmic vesicles (purple spheres) in pre-synaptic regions of intact terminals, compared with (B) cytoplasmic vesicles in pre-synaptic regions of damaged terminals, showed a reduction. (C) Synaptic ribbons (purple/violet) from pre-synaptic regions of intact terminals, (D) synaptic ribbons from pre-synaptic regions of damaged terminals. The number of vesicles appeared to be reduced, but the effect was variable. (E,F) tomographic slices showing vesicles (black arrow tethered at endoplasmic reticulum (ER), by filamentous tethers (white arrows). Inset shows reconstruction of ER and vesicle tether. (G) ER attached vesicles in pre-synaptic region of intact terminals. (H) ER attached vesicles in pre-synaptic region of damaged terminals. (I,J) tomographic slice showing synaptic membrane (SM) tethered vesicles, arrows as (E,F). (K) Synaptic membrane tethered vesicles at the synapse of an intact terminal. (L) Synaptic membrane tethered vesicles at the synapse of a damaged terminal. (M) Change in vesicle population number between intact (clear bars) and damaged (shaded bars). All populations showed a reduction, but this was only significant in the ER attached population. *P=0.015. [two-tailed Mann–Whitney U. *n*=6 terminals from four (intact) or three (damaged) animals. Error bars represent mean±s.d.]. Scale bars: (A–D,G,H) 200 nm, (K,L) 100 nm, (C,D) 50 nm, (E,F,I,J) 20 nm.

The changes to the membrane populations led to the hypothesis that the normal endocytic membrane recycling pathway was perturbed in the damaged terminal and therefore, that the number of synaptic vesicles available in the damaged terminal would be reduced. To assess this, the vesicle population of the pre-synaptic region was quantified. Vesicles in the pre-synaptic region were divided into several populations. Vesicles tethered at the synaptic membrane (the readily-releasable pool) and attached to the synaptic ribbon (the ribbon-attached pool) were subtracted from the vesicles free in cytoplasm. Also identifiable was a population of vesicles that were attached to the endoplasmic reticulum by filamentous tethers, similar to those we have previously described (Bullen et al., 2015). These were also subtracted from the total number of vesicles in the free cytoplasmic pool, and the populations were analysed separately. Cytoplasmic vesicles were reduced in the damaged terminals, although the population was quite variable between terminals (Fig. 6A–B, Table 2).

There were also fewer vesicles in the other vesicle populations. Ribbon attached vesicles showed a similar decrease, but as there were only three ribbons in each group the significance of this result could not be assessed (Fig. 6C,D,M, Table 2). Endoplasmic reticulum attached vesicles were significantly decreased (Fig. 6E–H,M, Table 2); whereas synaptic membrane tethered vesicles were reduced, but not significantly (Fig. 5I–L,M, Table 2). Vesicles were therefore reduced across all populations in the noise damaged terminals.

## DISCUSSION

Our previous work using 3D electron microscopy (Bullen et al., 2015) revealed an organised intracellular membrane system. The distribution of mitochondria was associated with this system, and membrane distribution was related to the distribution of synapses around the cell body of the IHC. This suggested a functional significance of intracellular organisation. The present work demonstrates that under conditions in which a significant proportion of afferent terminals are damaged or lost, despite a quite considerable change in cell shape as revealed by the 3D reconstructions, most markedly enlarged indentations around the basolateral pole, the overall, low resolution, intracellular organisation observed in normal intact cells was preserved and associated with the distribution of the remaining, seemingly intact terminals. This corresponds with maintenance of auditory thresholds. At the same time the higher resolution organisation in the presynaptic region of the IHC apposing an afferent terminal that showed noise-related damage was disrupted, whereas in the same cell it was preserved in the presynaptic region apposing a closely adjacent terminal that showed every feature consistent with an intact nerve ending. These observations would seem to imply mechanisms to maintain intracellular organisation in order to maintain function. The disruption to intracellular organisation that occurs with trauma-induced disruption is quite specific and highly localised, and implies an interaction across synapses between the nerve terminals and the immediate, local pre-synaptic region of the cell.

3D reconstruction demonstrated the severity and persistence of the effects of swelling of afferent terminals after noise exposure. Swollen terminals are sometimes an artefact associated with tissue fixation. We do not believe that fixation artefacts were a significant factor in the tissue changes observed in this study, due to the comparatively low rate of swollen terminals (<5%) identified in control cells fixed and prepared in parallel with noise damaged cells, the short time between euthanasia and fixation of tissue (<5 min), and the observation that swollen terminals were not obviously present outside the damaged region in noise damaged cochlea. Changes in the morphology of IHCs by the swelling of afferent terminals have been described as an acute phenomenon after trauma (Ruel et al., 2007). However, the persistence of tissue vacuolation 4 weeks after the noise insult was surprising; it may have been expected that the tissue would have recovered as had been observed after glutatoxic damage. In experiments where vacuolation of the tissue resulted from the application of agonist to the IHC synapses, recovery of normal basal pole shape occurred 3 h after application, and no empty spaces remained after 6 h (Ruel et al., 2007). In our experiments the basal pole remained severely distorted 4 weeks after application of damaging noise. Since the morphology of IHCs was consistent across noise-damaged cochleae, and was normal in control samples prepared in parallel with the noise damaged cochleae, the observed distortion of IHC shapes is unlikely to be an artefact of tissue preparation. However, as pointed out above, despite the deformation of the IHC body, organelle distribution was not significantly disturbed. Although previous evidence showed that low and medium SR fibres contact the cell on the modiolar side in the cat (Liberman, 1982; Liberman et al., 1990), no difference was observed in the number of damaged terminals on either side of the cell. The lack of a strong preference for damage on one side of the cells may be due to sample size, but may also reflect species specific differences. It has been reported in aging mice that synapse loss is not different on the modiolar-pillar axis (Stamataki et al., 2006), although a greater loss of LSR fibres was observed in ageing gerbils (Schmiedt et al., 1996). Also, light microscopy studies have suggested that there may be significant rearrangement of synaptic locations after noise exposure (Liberman et al., 2015). Such rearrangements may also account for the lack of a clear modiolar/pillar division in damaged terminals.

Two classes of noise affected terminals were present in the samples. ‘Lost’ terminals (where an indentation indicating an afferent bouton remained but a synaptic density and synaptic ribbon pair were not apparent) would seem to correlate well to the observation of the loss of staining for post-synaptic and ribbon proteins which is often used to quantify synaptic loss in light microscopy and upon which much of the assessment of terminal loss in hidden hearing loss has been based (see Kujawa and Liberman, 2015). However, the population of ‘damaged’ terminals, showing vacuolation of the afferent bouton but also the presence of a ribbon and synaptic density, suggest a potentially interesting population of terminals that may appear undamaged by light microscopy of immunostained terminals, but where the afferent bouton is in fact damaged. It is interesting to note that in this sample the distribution of lost terminals between the modiolar and pillar sides of the cell suggests an increased number of lost terminals on the modiolar side, although this result did not reach significance. If the distribution of low SR and high SR fibres is similar in the mouse to that previously described in the cat, this may suggest that low SR fibres on the modiolar side may lose both the terminal and synaptic specialisation in the IHC more quickly than other terminals. The presence of lost terminals without synaptic specialisation may also have implications for the fate of synapses after terminal damage: if synaptic proteins are down-regulated in the IHC after terminal loss, are synaptic components recovered at longer periods after noise exposure (with or without terminal regeneration)? Such questions have important consequences for assessment of terminal loss by immunostaining and light microscopy methods.

Although the distribution of organelles in the infranuclear region of noise exposed IHCs remained similar to unexposed cells, high resolution ultrastructural analysis of the pre-synaptic regions of these cells demonstrated the persistence of large membrane cisterns in the local pre synaptic region when the attaching afferent bouton showed damage. Evidence indicates that recycling of synaptic vesicles in IHCs predominantly occurs in the region local to the synapse (for review see Wichmann and Moser, 2015), and involves local membrane cisterns as demonstrated in frog saccule (Lenzi et al., 2002). In the pre-synaptic regions of intact terminals, these cisterns are small at rest. Larger and more numerous membrane cisterns in the pre-synaptic regions of damaged afferent terminals may represent cisterns arising from noise stimulated activity dependent bulk endocytosis (ADBE), which have not been recycled and have remained in the region. ADBE is a clathrin independent form of membrane retrieval employed by cells during periods of intense stimulation (Clayton et al., 2008; Royle and Lagnado, 2003), and occurs in IHCs under conditions of strong sustained stimulation (Neef et al., 2014). Under normal conditions large cisterns produced by ADBE would be resolved by the budding of new synaptic vesicles (Clayton and Cousin, 2009). This process is clathrin-dependent, and occurs within minutes of stimulation in the *Drosophila* neuromuscular junction (Heerssen et al., 2008), with a similar time course for resolution of large cisterns demonstrated in IHCs (Revelo et al., 2014).

The persistence of large cisterns in pre-synaptic regions of noise-damaged afferent terminals may indicate failure of the mechanisms governing the recycling of synaptic vesicles from membrane cisterns. This hypothesis is supported by several findings: the dramatic increase in uncoated membrane cisterns in noise damaged pre-synaptic regions; the concomitant decrease in the synaptic vesicle population; and the increase in the number and volume of coated and uncoated membrane in mixed membranes. The membrane coat was not unambiguously identified as clathrin, but is highly similar in appearance to the coating on invaginations at the plasma membrane, and to many previous images of clathrin coated vesicles (examples in Avinoam et al., 2015; Heymann et al., 2013). In addition, of the three classes of cytoplasmic coat protein complexes in eukaryotic cells (COPI, COPII and clathrin), only clathrin coated vesicles are known to bud from endosomes; the other types bud from the Golgi apparatus and ER respectively (Lee and Goldberg, 2010; Szul and Sztul, 2011).

In previous studies, the pre-synaptic vesicle population has been shown to vary depending on the spontaneous rate of the fibre, high SR fibres having on average lower vesicle numbers in the presynaptic region than fibres with low SR (Merchan-Perez and Liberman, 1996). If the population of intact fibres examined in this study were skewed towards the high SR fibres, due to the increased susceptibility of low SR fibres to noise damage, then the comparative reduction in vesicles in the presynaptic regions of damaged terminals compared to intact terminals may indicate an even more dramatic true decrease in vesicle numbers. In other nerve terminals clathrin coated vesicles are rapidly uncoated after fission from the plasma membrane (Milosevic et al., 2011), and models suggest that endocytic intermediates seen after strong stimulation are not the result of clathrin mediated endocytosis, but of bulk endocytosis (Saheki and De Camilli, 2012). Increased numbers and volumes of coated membrane in these mixed membranes therefore indicates delay or partial failure in vesicle recycling of large membrane cisterns at a late stage. Clathrin coated regions form, but are impeded in detaching from the membrane cisterns to form new vesicles.

It is not yet known how noise exposure may affect the process of synaptic recycling, but several possibilities may be proposed. Sustained presentation of intense stimulus to the IHCs may simply overwhelm the cell's ability to process the large membrane cisterns retrieved into synaptic vesicles. However it does not seem likely that cisterns would persist for 4 weeks after acoustic insult without further factors preventing their recycling into vesicles. Another possibility is that the cisterns do not persist, and that the membrane cisterns observed occur due to the exposure during the second ABR. However, considering the known short time course for the resolution of ADBE-derived cisterns in IHCs, and as both control and noise-damaged animals received the same stimulus and delay between completion of the ABR and fixation of the cochleae, this would still indicate perturbation of vesicle recycling in the noise damaged animals. Reduction in vesicle replenishment of the readily releasable pool has been described in mutants of the calcium sensing protein otoferlin, also an important mediator of exocytosis, although this may be due to otoferlin's interaction with vesicles at the synaptic ribbon (Duncker et al., 2013; Pangrsic et al., 2010). The synaptic ribbon has also been previously suggested to produce factors that promote vesicle fission (Kantardzhieva et al., 2013) and impairment of vesicle replenishment has been reported in mutants for the ribbon anchoring protein Bassoon (Frank et al., 2010). Loss or damage to the synaptic ribbon may therefore also be a factor in changes in vesicle recycling. Alternative mechanisms could involve specific protein complexes known to be important for the reformation of vesicles by clathrin dependent processes. Analogous to their functions at the plasma membrane, clathrin adapter proteins are likely to be important for clustering of synaptic vesicle proteins at cisterns produced by ADBE and the reformation of vesicles (Clayton and Cousin, 2009; Haucke and De Camilli, 1999). In IHCs, the clathrin adapter protein AP2 is known to interact with otoferlin. Vesicle fission is also known to be dependent on the GTPase dynamin (Mousavi et al., 2004). The failure of fission could therefore be due to failure of a specific protein or complex, or to changes to the energy state of the cell.

In the current study, it has been shown that pre-synaptic regions adjacent to damaged terminals have ultrastructural changes that may affect their function. To assess the importance of these changes to function two further questions must be addressed, do at least some damaged terminals recover sufficiently to continue to function, and do the changes in the pre-synaptic region persist after any such recovery. Recovery of damaged terminals is a debated phenomenon, with many reports suggesting terminals do not recover after loss (Kujawa and Liberman, 2015). However in some reports where recovery has been demonstrated, coding deficits at the auditory nerve fibres were present after recovery, affecting both intensity and temporal processing (Song et al., 2016). Recently, it has also been reported that IHC malfunction may be involved in changes after noise exposure (Mulders et al., 2018). Changes in vesicle recycling, persisting after synaptic repair, could affect these functions. A reduction in vesicle recycling may lead to a slower replenishment of the vesicle pool, and affect the intensity and temporal coding of responses. This question may be resolved by extending this work into other species and by further functional characterisation of recovered synapses.

This study demonstrates structural changes arising from noise exposure occurring within the IHC, particularly in the pre-synaptic regions of noise-damaged synapses. By using electron tomography and 3D reconstruction it was possible to understand the structural correlates of synaptic dysfunction and reveal specific changes that give mechanistic insights. The changes seen at noise-damaged synapses suggest a deficit in vesicle recycling, occurring during the regeneration of vesicles from large membrane cisterns. Further investigation of this effect may provide insights into vesicle recycling not only in IHCs but also other neuronal cells, and into the changes that may occur to synaptic coding even where synapses have recovered.

## MATERIALS AND METHODS

### Animals

Male CBA/Ca6 mice, aged between 8–10 weeks at exposure were used for all experiments. Control animals were age-matched littermates of exposed animals. All experiments were performed in accordance with the United Kingdom Animal (Scientific Procedures) Act of 1986.

### Noise exposure

Animals anaesthetised with ketamine/medetomidine were placed in a soundproof booth (40 dB attenuation) directly beneath the centre of a pre-calibrated speaker [Stage Line PA Horn Tweeter MHD-220/RD, frequency response flat (±2 dB) over the 8–16 kHz range] suspended 45 cm above the animals' head. Noise-exposed animals (*n*=7) were presented with 100 dB SPL octave band noise (8–16 kHz *n*=5, or 4-8 kHz *n*=2) for 2 hours. Noise stimuli were generated using an RX6 processor (Tucker Davis Technologies, TDT), attenuated (TDT PA5) and amplified (TDT SA2) as required. Control animals (*n*=4) were anaesthetised and placed in the soundproof booth for 2 hours, but were not exposed to noise (‘sham-exposure’). During noise and sham exposures, pedal reflex and breathing rate were monitored every 30 min and additional anaesthesia given upon indication.

### ABR

To assess the effects of noise exposure and the recovery of audiometric thresholds, auditory brainstem response (ABR) recordings were performed on all animals immediately prior to-, 1-day post-, and 4-weeks post-noise exposure. Animals were anaesthetised throughout recording with ketamine and medetomidine. Acoustic stimuli were presented free-field from a pre-calibrated speaker (TDT FF1, calibrated to ensure, after correction, the sound system frequency response was flat to within ±3 dB from 2–70 kHz), positioned at 45° to the animal's midline and 20 cm from the ipsilateral pinna; the contralateral ear was blocked using a foam ear-plug. Stimuli were either clicks (50 µs duration, 10–80 dB SPL in 5 dB steps) or tone-pips (5 ms total duration including 1.5 ms rise/fall time; 6, 8, 11, 16, 24, 32, and 48 kHz; 10–80 dB in 5 dB steps), delivered at a rate of 20/s. Stimuli were generated using a TDT RX6 processor, attenuated as needed (TDT PA5), and amplified (TDT SA2). ABR recordings were obtained using subdermal needle electrodes; one inserted at the vertex, and one each behind each pinna. Electrode signals were low-pass filtered (7.5 kHz cut-off frequency, 12 dB per octave) and recorded at 24 kHz sampling rate (TDT RA4LI, RA4PA and RX5). For analysis, ABR data were filtered using a bandpass filter (100–3000 Hz, 5th-order Butterworth filter), ABR thresholds were defined as the lowest sound level at which at least two of the deflections in the ABR waveform exceeded 2× the standard error of the background signal, as per Anderson and Linden (Anderson and Linden, 2016). Animals were euthanised on the same day as 4-week post exposure ABR measurements were taken.

### Fixation and decalcification

After euthanasia both auditory bullae from each animal were isolated and opened to expose the cochlea. Fixative (2.5% Glutaraldehyde in 0.01 M Cacodylate buffer) (Agar Scientific, UK) was injected gently into the cochlea via the round window, exiting via a hole made in the bone at the apex. Time between euthanasia and cochlea fixation was <5 min in all cases. The bullae were then immersed in fixative and fixation continued for 2 hours at room temperature. They were then decalcified in a solution of 4% EDTA in cacodylate buffer for 48 h at 4°C. The IHCs of several control animals were examined to check that fixation had not caused artefactual swelling of the nerve terminals. Tissue from noise-damaged animals was prepared in parallel with this control tissue.

### Transmission electron microscopy (TEM) and electron tomography (ET)

After fixation and decalcification samples were stained with Osmium Tetroxide (Agar Scientific) and Uranyl Acetate (Agar Scientific), dehydrated through a graded series of ethanols and polypropylene oxide before embedding. Samples were embedded in an Epon resin (TAAB 812, TAAB, UK). Blocks were cut to a mid-modiolar section, and sections were cut from here to allow selection of the approximate damage region for all blocks. For TEM, sections were cut at 100 nm on an ultramicrotome and placed on uncoated copper grids. Samples were imaged at 80 kV in a JEOL JEM-1200 EX II transmission electron microscope. For ET 200 nm sections on formvar-carbon coated copper grids (Agar Scientific) were imaged in a JEOL JEM-2100 microscope operating at 200 kV. Montage (2×2) dual axis tomogram tilt series were collected at 1° increments for an axis of −60–60° and a pixel size of 0.371 nm.

### Serial block face scanning electron microscopy (SBF-SEM)

After fixation and decalcification samples were incubated overnight in a solution of 0.05% tannic acid, and were then prepared for SBF-SEM following the method of Denk and Horstmann (2004). Briefly, samples were incubated in a 3% solution of potassium ferranocyanate and 2% osmium tetroxide, followed by 10% thiocarbohydrazide, and then 2% osmium tetroxide followed by 1% uranyl acetate. Samples then underwent en-bloc lead aspartate staining before dehydration by graded ethanols and polypropylene oxide, and embedding in Epon resin. For imaging, samples were cut from mid-modiolar sections into blocks containing the frequency region of interest (approximately 32 kHz) and mounted on cryo-pins (Leica, Germany). Samples were imaged in a Gatan 3View XP system on a JEOL JSM-7100F scanning electron microscope cutting at successive 50 nm depths and using an x,y pixel size of 19–25 nm.

### Image reconstruction, segmentation and modelling

Reconstruction and alignment of ET and SBF-SEM image data was carried out using etomo, part of the IMOD programme suite (Kremer et al., 1996). Tomograms were reconstructed using either weighted back projection or serial iterative reconstruction technique. Both datasets were segmented in IMOD, using the 3DMOD programme. For SBF-SEM images cells were reconstructed by manual segmentation, with contours every 250 nm. Mesh data was automatically interpolated between the contours. Afferent boutons and synaptic ribbons were represented by spheres of fixed size. Division of cells into modiolar and pillar hemispheres with a dynamic centre line was carried out as described in (Bullen et al., 2015). For ET, objects were reconstructed using semi-automated ‘livewire’ segmentation, with contours every 2.2 nm. Mesh data were automatically interpolated between contours. Quantitative data were extracted from the models using the imodinfo script. Stereology data was analysed using bespoke python scripts (see Bullen et al., 2015). These scripts may be obtained from the authors.

### Experimental design and statistical analysis

Visual analysis suggested a very large effect size. A sample size calculation using a Cohen's *d* of 2 in a two-tailed Mann–Whitney *U*-test using the G*Power software (Faul et al., 2007) suggested a sample size of six per group would be sufficient. This was also considered practical in terms of the time required for reconstruction of 3D volumes. For whole cell analysis several control cochleae were examined, and six adjacent cells were reconstructed from a single control animal to ensure that variations in fixation between adjacent cells (and therefore, no selection for ‘good’ cells) was present in the analysis. For noise exposed animals, six cells in total from two noise exposed animals (four from animal A, two from animal B) that were prepared separately several weeks apart were used. The animals had been noise damaged with a noise band between 8–16 kHz and cochleae were examined in the 32–40 kHz region. For electron tomography of pre-synaptic regions, six intact terminals were selected from control (three) and noise exposed (three) animals, using a total of four animals. Six noise damaged terminals were selected from 4–8 kHz (three) and 8–16 kHz (three) using a total of three animals.

Statistical tests were carried out in SPSS (IBM, USA). Due to the small sample size, normality of the sample population could not be assumed. Significance testing was carried out using the Mann–Whitney *U*-test, reporting the two-tailed exact significance (accounting for ties). It should be noted that for the *n* numbers used (*n*=6 subjects per group, unless otherwise specified) the lower limit of significance that may be reported by this test is *P*=0.002. Mean is reported with sample standard deviation (s.d.) unless otherwise stated.

## References

[BIO038547C1] Anderson L. A. and Linden J. F. (2016). Mind the gap: two dissociable mechanisms of temporal processing in the auditory system. *J. Neurosci.* 36, 1977-1995. 10.1523/JNEUROSCI.1652-15.2016 26865621 PMC4748080

[BIO038547C2] Avinoam O., Schorb M., Beese C. J., Briggs J. A. and Kaksonen M. (2015). ENDOCYTOSIS. Endocytic sites mature by continuous bending and remodeling of the clathrin coat. *Science* 348, 1369-1372. 10.1126/science.aaa9555 26089517

[BIO038547C3] Bullen A., West T., Moores C., Ashmore J., Fleck R. A., MacLellan-Gibson K. and Forge A. (2015). Association of intracellular and synaptic organization in cochlear inner hair cells revealed by 3D electron microscopy. *J. Cell Sci.* 128, 2529-2540. 10.1242/jcs.170761 26045447 PMC4510854

[BIO038547C4] Clayton E. L. and Cousin M. A. (2009). The molecular physiology of activity-dependent bulk endocytosis of synaptic vesicles. *J. Neurochem.* 111, 901-914. 10.1111/j.1471-4159.2009.06384.x 19765184 PMC2871311

[BIO038547C5] Clayton E. L., Evans G. J. O. and Cousin M. A. (2008). Bulk synaptic vesicle endocytosis is rapidly triggered during strong stimulation. *J. Neurosci.* 28, 6627-6632. 10.1523/JNEUROSCI.1445-08.2008 18579735 PMC2588494

[BIO038547C6] Costalupes J. A., Young E. D. and Gibson D. J. (1984). Effects of continuous noise backgrounds on rate response of auditory nerve fibers in cat. *J. Neurophysiol.* 51, 1326-1344. 10.1152/jn.1984.51.6.1326 6737033

[BIO038547C7] Denk W. and Horstmann H. (2004). Serial block-face scanning electron microscopy to reconstruct three-dimensional tissue nanostructure. *PLoS Biol.* 2, e329 10.1371/journal.pbio.0020329 15514700 PMC524270

[BIO038547C8] Duncker S. V., Franz C., Kuhn S., Schulte U., Campanelli D., Brandt N., Hirt B., Fakler B., Blin N., Ruth P. et al. (2013). Otoferlin couples to clathrin-mediated endocytosis in mature cochlear inner hair cells. *J. Neurosci.* 33, 9508-9519. 10.1523/JNEUROSCI.5689-12.2013 23719817 PMC3676539

[BIO038547C9] Faul F., Erdfelder E., Lang A.-G. and Buchner A. (2007). G*Power 3: a flexible statistical power analysis program for the social, behavioral, and biomedical sciences. *Behav. Res. Methods* 39, 175-191. 10.3758/BF03193146 17695343

[BIO038547C10] Frank T., Rutherford M. A., Strenzke N., Neef A., Pangršič T., Khimich D., Fejtova A., Gundelfinger E. D., Liberman M. C., Harke B. et al. (2010). Bassoon and the synaptic ribbon organize Ca(2)+ channels and vesicles to add release sites and promote refilling. *Neuron* 68, 724-738. 10.1016/j.neuron.2010.10.027 21092861 PMC3005353

[BIO038547C11] Fuchs P. A., Lehar M. and Hiel H. (2014). Ultrastructure of cisternal synapses on outer hair cells of the mouse cochlea. *J. Comp. Neurol.* 522, 717-729. 10.1002/cne.23478 24122766 PMC4474150

[BIO038547C12] Furman A. C., Kujawa S. G. and Liberman M. C. (2013). Noise-induced cochlear neuropathy is selective for fibers with low spontaneous rates. *J. Neurophysiol.* 110, 577-586. 10.1152/jn.00164.2013 23596328 PMC3742994

[BIO038547C13] Haucke V. and De Camilli P. (1999). AP-2 recruitment to synaptotagmin stimulated by tyrosine-based endocytic motifs. *Science* 285, 1268-1271. 10.1126/science.285.5431.1268 10455054

[BIO038547C14] Heerssen H., Fetter R. D. and Davis G. W. (2008). Clathrin dependence of synaptic-vesicle formation at the Drosophila neuromuscular junction. *Curr. Biol.* 18, 401-409. 10.1016/j.cub.2008.02.055 18356056 PMC2699046

[BIO038547C15] Hesse L. L., Bakay W., Ong H.-C., Anderson L., Ashmore J., McAlpine D., Linden J. and Schaette R. (2016). Non-monotonic relation between noise exposure severity and neuronal hyperactivity in the auditory midbrain. *Front. Neurol.* 7, 133 10.3389/fneur.2016.00133 27625631 PMC5004570

[BIO038547C16] Heymann J. B., Winkler D. C., Yim Y.-I., Eisenberg E., Greene L. E. and Steven A. C. (2013). Clathrin-coated vesicles from brain have small payloads: a cryo-electron tomographic study. *J. Struct. Biol.* 184, 43-51. 10.1016/j.jsb.2013.05.006 23688956 PMC3796050

[BIO038547C17] Kantardzhieva A., Liberman M. C. and Sewell W. F. (2013). Quantitative analysis of ribbons, vesicles, and cisterns at the cat inner hair cell synapse: correlations with spontaneous rate. *J. Comp. Neurol.* 521, 3260-3271. 10.1002/cne.23345 23787810 PMC4309284

[BIO038547C18] Kremer J. R., Mastronarde D. N. and McIntosh J. R. (1996). Computer visualization of three-dimensional image data using IMOD. *J. Struct. Biol.* 116, 71-76. 10.1006/jsbi.1996.0013 8742726

[BIO038547C19] Kujawa S. G. and Liberman M. C. (2009). Adding insult to injury: cochlear nerve degeneration after “temporary” noise-induced hearing loss. *J. Neurosci.* 29, 14077-14085. 10.1523/JNEUROSCI.2845-09.2009 19906956 PMC2812055

[BIO038547C20] Kujawa S. G. and Liberman M. C. (2015). Synaptopathy in the noise-exposed and aging cochlea: primary neural degeneration in acquired sensorineural hearing loss. *Hear. Res.* 330, 191-199. 10.1016/j.heares.2015.02.009 25769437 PMC4567542

[BIO038547C21] Lee C. and Goldberg J. (2010). Structure of coatomer cage proteins and the relationship among COPI, COPII, and clathrin vesicle coats. *Cell* 142, 123-132. 10.1016/j.cell.2010.05.030 20579721 PMC2943847

[BIO038547C22] Lenzi D., Crum J., Ellisman M. H. and Roberts W. M. (2002). Depolarization redistributes synaptic membrane and creates a gradient of vesicles on the synaptic body at a ribbon synapse. *Neuron* 36, 649-659. 10.1016/S0896-6273(02)01025-5 12441054

[BIO038547C23] Liberman M. C. (1982). Single-neuron labeling in the cat auditory nerve. *Science* 216:1239-1241. 10.1126/science.7079757 7079757

[BIO038547C24] Liberman M. C., Dodds L. W. and Pierce S. (1990). Afferent and efferent innervation of the cat cochlea - quantitative-analysis with light and electron-microscopy. *J. Comp. Neurol.* 301, 443-460. 10.1002/cne.903010309 2262601

[BIO038547C25] Liberman L. D., Suzuki J. and Liberman M. C. (2015). Dynamics of cochlear synaptopathy after acoustic overexposure. *J. Assoc. Res. Otolaryngol.* 16, 205-219. 10.1007/s10162-015-0510-3 25676132 PMC4368657

[BIO038547C26] Lin H. W., Furman A. C., Kujawa S. G. and Liberman M. C. (2011). Primary neural degeneration in the Guinea pig cochlea after reversible noise-induced threshold shift. *J. Assoc. Res. Otolaryngol.* 12, 605-616. 10.1007/s10162-011-0277-0 21688060 PMC3173555

[BIO038547C50] Mann H. B. and Whitney D. R. (1947). On a Test of Whether One of 2 Random Variables Is Stochastically Larger Than the Other. *Ann Math Stat.* 18, 50-60. 10.1214/aoms/1177730491

[BIO038547C27] Matsubara A., Laake J. H., Davanger S., Usami S. and Ottersen O. P. (1996). Organization of AMPA receptor subunits at a glutamate synapse: a quantitative immunogold analysis of hair cell synapses in the rat organ of Corti. *J. Neurosci.* 16, 4457-4467. 10.1523/JNEUROSCI.16-14-04457.1996 8699256 PMC6578857

[BIO038547C28] Merchan-Perez A. and Liberman M. C. (1996). Ultrastructural differences among afferent synapses on cochlear hair cells: correlations with spontaneous discharge rate. *J. Comp. Neurol.* 371, 208-221. 10.1002/(SICI)1096-9861(19960722)371:2<208::AID-CNE2>3.0.CO;2-6 8835727

[BIO038547C29] Milosevic I., Giovedi S., Lou X., Raimondi A., Collesi C., Shen H., Paradise S., O'Toole E., Ferguson S., Cremona O. et al. (2011). Recruitment of endophilin to clathrin-coated pit necks is required for efficient vesicle uncoating after fission. *Neuron* 72, 587-601. 10.1016/j.neuron.2011.08.029 22099461 PMC3258500

[BIO038547C30] Mousavi S. A., Malerød L., Berg T. and Kjeken R. (2004). Clathrin-dependent endocytosis. *Biochem. J.* 377, 1-16. 10.1042/bj20031000 14505490 PMC1223844

[BIO038547C31] Mulders W. H. A. M., Chin I. L. and Robertson D. (2018). Persistent hair cell malfunction contributes to hidden hearing loss. *Hear. Res.* 361, 45-51. 10.1016/j.heares.2018.02.001 29477697

[BIO038547C32] Neef J., Jung S. Y., Wong A. B., Reuter K., Pangršič T., Chakrabarti R., Kügler S., Lenz C., Nouvian R., Boumil R. M. et al. (2014). Modes and regulation of endocytic membrane retrieval in mouse auditory hair cells. *J. Neurosci.* 34, 705-716. 10.1523/JNEUROSCI.3313-13.2014 24431429 PMC3891952

[BIO038547C33] Pangršič T., Lasarow L., Reuter K., Takago H., Schwander M., Riedel D., Frank T., Tarantino L. M., Bailey J. S., Strenzke N. et al. (2010). Hearing requires otoferlin-dependent efficient replenishment of synaptic vesicles in hair cells. *Nat. Neurosci.* 13, 869-876. 10.1038/nn.2578 20562868

[BIO038547C34] Puel J.-L., Ruel J., Gervais d'Aldin C. and Pujol R. (1998). Excitotoxicity and repair of cochlear synapses after noise-trauma induced hearing loss. *Neuroreport* 9, 2109-2114. 10.1097/00001756-199806220-00037 9674603

[BIO038547C35] Revelo N. H., Kamin D., Truckenbrodt S., Wong A. B., Reuter-Jessen K., Reisinger E., Moser T. and Rizzoli S. O. (2014). A new probe for super-resolution imaging of membranes elucidates trafficking pathways. *J. Cell Biol.* 205, 591-606. 10.1083/jcb.201402066 24862576 PMC4033769

[BIO038547C36] Robertson D. (1983). Functional significance of dendritic swelling after loud sounds in the guinea pig cochlea. *Hear. Res.* 9, 263-278. 10.1016/0378-5955(83)90031-X 6841283

[BIO038547C37] Royle S. J. and Lagnado L. (2003). Endocytosis at the synaptic terminal. *J. Physiol.* 553, 345-355. 10.1113/jphysiol.2003.049221 12963793 PMC2343565

[BIO038547C38] Ruel J., Wang J., Rebillard G., Eybalin M., Lloyd R., Pujol R. and Puel J.-L. (2007). Physiology, pharmacology and plasticity at the inner hair cell synaptic complex. *Hear. Res.* 227, 19-27. 10.1016/j.heares.2006.08.017 17079104

[BIO038547C39] Saheki Y. and De Camilli P. (2012). Synaptic vesicle endocytosis. *Cold Spring Harb Perspect Biol.* 4, a005645 10.1101/cshperspect.a005645 22763746 PMC3428771

[BIO038547C40] Schaette R. and McAlpine D. (2011). Tinnitus with a normal audiogram: physiological evidence for hidden hearing loss and computational model. *J. Neurosci.* 31, 13452-13457. 10.1523/JNEUROSCI.2156-11.2011 21940438 PMC6623281

[BIO038547C41] Schmiedt R. A., Mills J. H. and Boettcher F. A. (1996). Age-related loss of activity of auditory-nerve fibers. *J. Neurophysiol.* 76, 2799-2803. 10.1152/jn.1996.76.4.2799 8899648

[BIO038547C51] Schnee M. E., Lawton D. M., Furness D. N., Benke T. A. and Ricci A. J. (2005). Auditory hair cell-afferent fiber synapses are specialized to operate at their best frequencies. *Neuron.* 47, 243-254. 10.1016/j.neuron.2005.06.004 16039566

[BIO038547C42] Shi L., Liu L., He T., Guo X., Yu Z., Yin S. and Wang J. (2013). Ribbon synapse plasticity in the cochleae of Guinea pigs after noise-induced silent damage. *PLoS ONE* 8, e81566 10.1371/journal.pone.0081566 24349090 PMC3857186

[BIO038547C43] Shi L., Liu K., Wang H., Zhang Y., Hong Z., Wang M., Wang X., Jiang X. and Yang S. (2015). Noise induced reversible changes of cochlear ribbon synapses contribute to temporary hearing loss in mice. *Acta Otolaryngol.* 135, 1093-1102. 10.3109/00016489.2015.1061699 26139555

[BIO038547C44] Shi L., Chang Y., Li X., Aiken S. J., Liu L. and Wang J. (2016). Coding deficits in noise-induced hidden hearing loss may stem from incomplete repair of ribbon synapses in the cochlea. *Front. Neurosci.* 10, 231 10.3389/fnins.2016.00231 27252621 PMC4879136

[BIO038547C45] Song Q., Shen P., Li X., Shi L., Liu L., Wang J., Yu Z., Stephen K., Aiken S., Yin S. et al. (2016). Coding deficits in hidden hearing loss induced by noise: the nature and impacts. *Sci. Rep.* 6, 25200 10.1038/srep25200 27117978 PMC4846864

[BIO038547C46] Stamataki S., Francis H. W., Lehar M., May B. J. and Ryugo D. K. (2006). Synaptic alterations at inner hair cells precede spiral ganglion cell loss in aging C57BL/6J mice. *Hear. Res.* 221, 104-118. 10.1016/j.heares.2006.07.014 17005343

[BIO038547C47] Szul T. and Sztul E. (2011). COPII and COPI traffic at the ER-Golgi interface. *Physiology* 26, 348-364. 10.1152/physiol.00017.2011 22013193

[BIO038547C48] Wichmann C. and Moser T. (2015). Relating structure and function of inner hair cell ribbon synapses. *Cell Tissue Res.* 361, 95-114. 10.1007/s00441-014-2102-7 25874597 PMC4487357

